# Blocking protein-protein interactions: the identification of repetitive turn structures as basis for inhibitor building blocks

**DOI:** 10.1186/1758-2946-4-S1-O21

**Published:** 2012-05-01

**Authors:** Oliver Koch

**Affiliations:** 1 Intervet Innovation GmbH, Schwabenheim, Germany; 2 MOLISA GmbH, Magdeburg, Germany

## 

Mimetics of secondary structure elements are one promising approach in the design of protein-protein interaction inhibitors, since secondary structure elements are very important recognition motifs in protein-protein interfaces. In helices and turns, the protein backbone provides a scaffold to present the sidechains in the correct orientation for the three-dimensional interaction motif. For both, scaffolds are known that resemble these backbone conformations and can be decorated with sidechains in the right position for mimicking the interaction motif [1]. Benzodiazepines are one example for a successful mimetic of β-turn structures.

However, identifying small chemical scaffolds that mimic turn structures is rather complicated. Turns are irregular structures with a wider variety of possible backbone conformations [2] and for each group of conformations a different scaffold is needed. Furthermore, turn structures are generally not included in analysis of protein-protein interfaces. Due to a lack of information in publicly available databases, regions of the protein chains that are outside helices and β-sheets are generally considered as non-regular structural elements. These non-regular structural elements in proteins are by now almost completely classified as turn structures and available via Secbase for data mining approaches [3].

The results of an exhaustive analysis of turn structures involved in protein-protein interfaces will be presented and the impact on the design of secondary structure element mimetics will be discussed. This is of particular interest since the secondary structure space of protein-protein interfaces is limited and similar interfaces with respect to secondary structure elements exists within proteins showing different overall folds and function [4]. The identification of repetitive turn structures is therefore a valuable approach to predict polypharmacology or identify backbone conformations that could easily be replaced by mimetic building blocks [4]. The decoration of these building blocks with the needed functional sidechain is a good starting point for protein-protein interface inhibitors.
